# Metabolomic Profiling of Bile Acids in an Experimental Model of Prodromal Parkinson’s Disease

**DOI:** 10.3390/metabo8040071

**Published:** 2018-10-31

**Authors:** Stewart F. Graham, Nolwen L. Rey, Zafer Ugur, Ali Yilmaz, Eric Sherman, Michael Maddens, Ray O. Bahado-Singh, Katelyn Becker, Emily Schulz, Lindsay K. Meyerdirk, Jennifer A. Steiner, Jiyan Ma, Patrik Brundin

**Affiliations:** 1 Beaumont Health, 3811 W. 13 Mile Road, Royal Oak, MI 48073, USA; Zafer.Ugur@beaumont.org (Z.U.); Ali.yilmaz@beaumont.org (A.Y.); mmaddens@beaumont.edu (M.M.); Ray.Bahado-Singh@beaumont.org (R.O.B.-S.); 2 Oakland University-William Beaumont School of Medicine, Rochester, MI 48309, USA; 3 Center for Neurodegenerative Science, Van Andel Research Institute, Grand Rapids, MI 49503, USA; Nolwen.REY@cnrs.fr (N.L.R.); Katelyn.Becker@vai.org (K.B.); emily.schulz@vai.org (E.S.); Lindsay.Meyerdirk@vai.org (L.K.M.); Jennifer.Steiner@vai.org (J.A.S.); Jiyan.Ma@vai.org (J.M.); Patrik.Brundin@vai.org (P.B.); 4 University of Michigan, Ann Arbor, MI 48109, USA; ebsherm@umich.edu

**Keywords:** prodromal Parkinson’s disease, bile acids, mass spectrometry, biomarkers, α-synuclein aggregates

## Abstract

For people with Parkinson’s disease (PD), considered the most common neurodegenerative disease behind Alzheimer’s disease, accurate diagnosis is dependent on many factors; however, misdiagnosis is extremely common in the prodromal phases of the disease, when treatment is thought to be most effective. Currently, there are no robust biomarkers that aid in the early diagnosis of PD. Following previously reported work by our group, we accurately measured the concentrations of 18 bile acids in the serum of a prodromal mouse model of PD. We identified three bile acids at significantly different concentrations (*p* < 0.05) when mice representing a prodromal PD model were compared with controls. These include ω-murichoclic acid (MCAo), tauroursodeoxycholic acid (TUDCA) and ursodeoxycholic acid (UDCA). All were down-regulated in prodromal PD mice with TUDCA and UDCA at significantly lower levels (17-fold and 14-fold decrease, respectively). Using the concentration of three bile acids combined with logistic regression, we can discriminate between prodromal PD mice from control mice with high accuracy (AUC (95% CI) = 0.906 (0.777–1.000)) following cross validation. Our study highlights the need to investigate bile acids as potential biomarkers that predict PD and possibly reflect the progression of manifest PD.

## 1. Introduction

Parkinson’s Disease (PD) is a common, long-term neurodegenerative disease. Adjusting for age and gender, the incidence of PD has been estimated to affect 1 in every 100 people over the age of 60 [1]. PD motor symptoms are believed to originate from striatal dopamine loss which occurs due to the death of dopaminergic neurons in the substantia nigra pars compacta (SNpc). The loss of dopaminergic neurons in the SNpc is the hallmark indicator for the post-mortem diagnosis of PD [2]. Lewy bodies and Lewy neurites, composed mainly of misfolded α-synuclein (α-syn) protein also feature in PD brains. Clinical diagnosis of PD is based on several criteria including bradykinesia in combination with rigidity, resting tremor, or both and response to dopaminergic drugs [3]. In addition to the classical motor symptoms, a wide range of non-motor symptoms and signs are apparent in PD patients [4], some of which are already present long before the onset of motor symptoms, in the PD prodrome [5]. However, misdiagnosis is common in the prodromal phase, when a potential disease-modifying treatment is thought to be most effective [6,7]. Currently, no robust biomarkers for early and more precise diagnosis of PD exist [8] and as several new potentially disease-modifying treatments emerge this is becoming a major unmet medical need [6,9]. 

In a previous study by our group, we identified Bile Acid metabolism as one of the major biochemical pathways to be perturbed in the brain of a mouse model of prodromal PD [10]. Bile acids are molecules derived from cholesterol in hepatocytes and are used to emulsify fats in the small intestine and promote fat digestion and absorption [11,12]. In addition to their role in lipid digestion and absorption, bile acids function as signaling molecules, participating as ligands in both membrane-bound receptors and nuclear hormone receptors [13,14]. It has been reported that certain bile acids, including ursodeoxycholic acid (UDCA) and tauroursodeoxycholic acid (TUDCA) can pass the blood–brain barrier [14] with their presence also being noted in cerebrospinal fluid (CSF), plasma, urine, and serum [15,16,17,18]. To date, several reports implicate bile acids in neurodegenerative diseases and suggest a possible role in modulating neuronal proliferation. One such study links statistically significant increases in levels of deoxycholic acid (DCA), glycodeoxycholic acid (GDCA), and lithocholic acid (LCA) in plasma, to Alzheimer’s disease and mild cognitive impairment [19]. Abdelkader et al. observed a neuroprotective effect from administration of UDCA on a murine rotenone model of PD [20]. Further, it has been reported that cholic acid is a ligand for liver X receptors which promote ventral midbrain neurogenesis and cell survival [21]. Bile acids have also been reported to be potential biomarkers of other neurodegenerative diseases including Alzheimer’s disease (AD) [22,23,24]. 

In the current study, we accurately measured the concentrations of 18 bile acids in the serum of a prodromal mouse model of PD. Following on from our previous metabolomics work using this model, we believe that bile acids may prove to be essential for the development of a robust biomarker panel capable of accurately diagnosing PD.

## 2. Results

### 2.1. Univariate Analysis

To investigate bile acids in a model of prodromal PD, we used a mouse model previously developed by our group which consists of WT mice injected with α-syn fibrils into the olfactory bulb [7,10]. The injection of α-syn fibrils leads to the propagation of α-syn aggregates throughout several interconnected regions in the brain. The progressive spreading of α-synucleinopathy shows many similarities with that which has been suggested to occur in PD [23,25,26,27]. Using mass spectrometry, we analyzed the serum of the α-syn fibrils-injected mice (PFF mice) and of α-syn monomers-injected mice (HuMonomers mice; controls), collected 3 months post injection.

Of the 18 bile acids profiled, all were within the limits of detection and quantification. Of these, we found three to be significantly perturbed in PFF mice compared to HuMonomers mice: Omega-murichoclic acid (MCAo), tauroursodeoxycholic acid (TUDCA) and ursodeoxycholic acid (UDCA) (Table 1). Of the three bile acids, we found UDCA and its taurine conjugated form TUDCA to be extremely decreased (17- and 14-fold, respectively) in the mice injected with PFFs.

**Figure 1:**
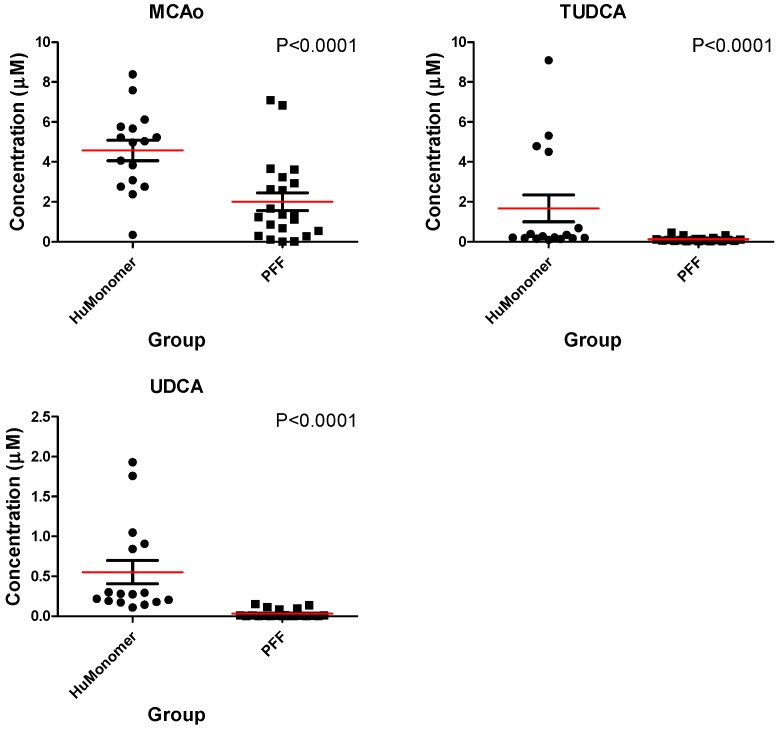
The mean distribution (±SEM) for each of the three significantly different bile acids between mice injected with HuMonomers and PFFs.

Figure 1 displays the Box and Whisker plots for the top three significantly different (*p* < 0.05; FDR < 0.05) metabolites in both the HuMonomer- and PFF-injected mice. As is evident from the plots, all are at significantly lower concentrations in PFF-injected mice.

### 2.2. Logistic Regression Analysis

Using the concentrations of taurolithocholic acid (TLCA), glycochenodeoxycholic acid (GCDCA) and TUDCA, we developed a diagnostic algorithm capable of accurately differentiating between HuMonomer- and PFF-injected mice with 91.4% accuracy following 100-fold cross validations.

logit(P) = log(P/(1 − P)) = −0.893 + 11.152 TLCA + 8.917 GCDCA − 18.221 TUDCA

where P is Pr(y = 1|x). The best threshold (or Cutoff) for the predicted P is 0.52. Original Label: 0/1 --> Labels in Logistic Regression: 0/1 Note) The class/response value is recommended as (Case: 1 and Control: 0).

**Figure 2:**
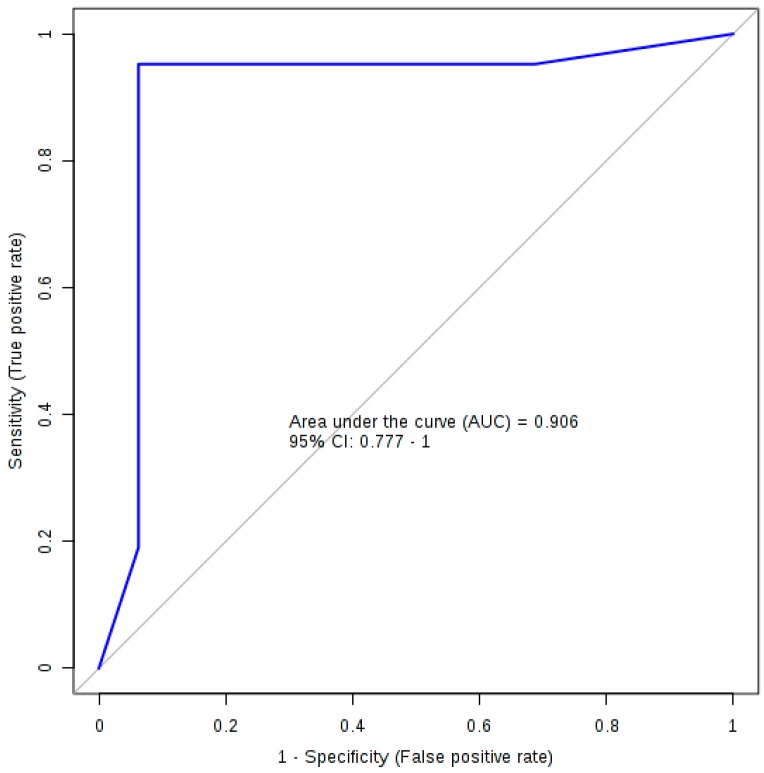
The ROC plot for the logistic regression diagnostic algorithm.

Table 2 lists the summary of each feature used to produce the diagnostic algorithm. Table 3 details the performance values of the logistic regression model following 10-fold cross validation with Figure 2 displaying the ROC plot for said model. The model was significant following 1000-permutation tests with *p* = 0.003. Figure 2 displays the ROC curve for the logistic regression analysis following 10-fold cross validation.

## 3. Discussion

This is the first study to accurately quantify bile acids from the serum of a validated mouse model of prodromal PD. Our study was primarily driven by the results from a previous study by our group [10]. In total, we profiled 18 bile acids of which only three were found to be statistically significantly different in PFF mice when compared with HuMonomer controls (*p* < 0.05). All three were found to be significantly decreased in PFF mouse serum, with TUDCA and UDCA at 14- and 17-fold lower concentrations, respectively. 

Using the concertation of three bile acids (TLCA, GCDCA and TUDCA), we developed a predictive model capable of differentiating between PFF mice and HuMonomer controls with an AUC (95 % CI) = 0.906 (0.777–1.00) with high sensitivity and specificity values (0.952 (0.952–1.000) and 0.938 (0.819–1.000), respectively) following cross validation. This eclipses work previously reported by our group in which we report a predictive logistic regression model developed using the concentration of three phosphocholines and trans-4-hrdroxyproline [10]. This previous model achieved an AUC (95% CI) = 0.836 (0.696−0.9777) high sensitivity and specificity values (0.800 (0.800−0.975) and 0.889 (0.744−1.00), respectively); however, following cross validation, those results are less precise than what we report herein. 

**Figure 3:**
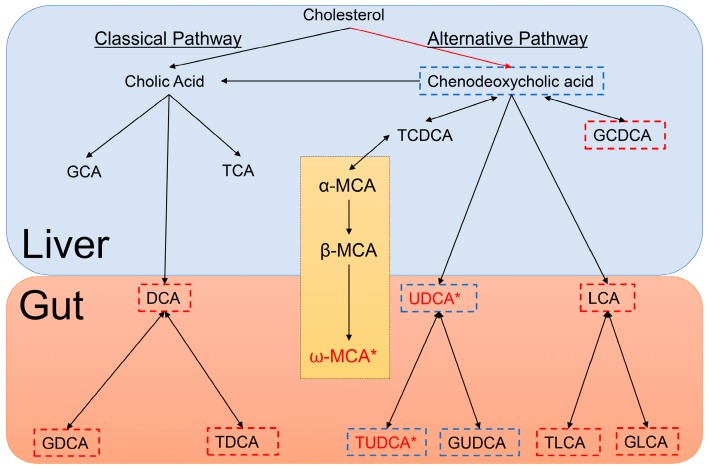
Depiction of Bile Acid Metabolism in the liver and gut of mice. Bile acids outlined in blue are neuroprotective, bile acids outlined in red are cytotoxic and those bile acids in red with an accompanying asterisk are statistically significantly different between HuMonomer- and PFF-injected mice. The section detailing Muricholic acid (MCA) only occurs in mice.

Bile acids play pivotal roles in many physiological and pathological activities which include acting as signaling molecules that regulate lipid, glucose and energy metabolism [28]; however, very little is known about the molecular mechanisms of bile acids in the central nervous system [29]. It has, however, been shown that following primary bile acid synthesis in the liver, bile acids are subsequently secreted into the gut where they are modified by the intestinal bacteria to produce secondary bile acids. These can be further modified in the liver or gut and may be conjugated with glycine or taurine [30]. Figure 3 displays a simplified depiction of the biochemistry. In Figure 3, we show which bile acids have been reported as being cytotoxic and neuroprotective [31,32]. Of the neuroprotective bile acids measured in this study, UDCA and TUDCA were found to be at markedly lower concentrations in the serum of PFF mice as compared to controls (17-fold and 14-fold, respectively). UDCA and TUDCA are secondary bile acids, produced in the gut and not in the liver. They have been reported to have neuroprotective effects in the brain, functioning partly as chaperones, decreasing the formation of toxic aggregates in protein folding disorders [33,34]. Further, they have also been reported to reduce reactive oxygen species formation [35], inhibit apoptosis [36] and prevent mitochondrial dysfunction [37].

A recent emerging and exciting concept in health and disease is the ability of the guts microbiota to communicate with the brain and subsequently modulate behavior [38]. This bidirectional signaling axis between the gut and the brain is believed to be essential for conserving homeostasis which is regulated at the hormonal, immunological and neuronal levels (central and enteric nervous systems) [38]. While a lot of attention has been placed on the gut microbiome and neurodegenerative diseases, most of the reported studies have focused on the gut as being the driver. In this study, we show that by inducing α-synucleinopathy in the brain with PFFs to mirror what is observed in prodromal PD, we see a significant decrease in the concentrations of secondary bile acids which have neuroprotective properties. As depicted in Figure 3, the production of these secondary, neuroprotective bile acids only occurs in the gut by intestinal bacteria. So, is the formation of the α-syn aggregates in the brain directly affecting the PFF mouse gut bacteria and the formation of secondary bile acids deemed neuroprotective? Or is it possible that these neuroprotective bile acids are being degraded faster in the prodromal PD brain due to the developing α-synucleinopathy which subsequently leads to lower blood concentrations? Both hypotheses need further exploration in the future.

We report, for the first time, a bile acid biomarker panel capable of identifying mice with developing α-synucleinopathy. Using bile acids as biomarkers is a marked improvement on our previous metabolomics work and highlights the potential of bile acids for the prediction of those patients at greatest risk of developing PD, particularly in the prodromal phase when a treatment aiming at slowing disease progression is potentially most effective and might even delay the onset of motor symptoms [8,9]. Further, our results demonstrate a potential novel therapeutic area for prodromal PD and developing α-synucleinopathy which needs future exploration. More work is required to verify these initial hypotheses, using mouse models and, most importantly, large clinical cohorts of people who exhibit several signs of prodromal PD. 

## 4. Materials and Methods

### 4.1. Animals

Under 12 h light/12 h dark cycles, C57Bl/6J mice (Jackson Laboratory) were housed four to five per cage with *ad libitum* access to food and water. As previously described by our group, all procedures relating to the animals followed The Guide for Care and Use of Laboratory Animals (National Research Council) and were validated by the Van Andel Research Institute’s Institutional Animal Care and Use Committee (Animal Use Protocols 14-01-001 and 16-12-033).

### 4.2. Purification of Recombinant α-syn, Assembly of Preformed Fibrils and Stereotactic Injections

Recombinant α-syn purification, assembly of the fibrils and stereotactic injections were previously described by our group [7,10,39]. In brief, we cultured BL21 *E. coli* and induced them to express human α-syn. The bacteria were then pelleted, and lysed by sonication. We boiled the lysate for 10 min and collected the supernatant after centrifugation. The supernatant was then dialyzed overnight in 10 mM Tris, pH 7.5, 50 mM NaCl, and 1 mM EDTA. The lysate was then purified by chromatographic separation using a Superdex 200 Column (GE Healthcare Life Sciences, Marlborough, MA, USA) and a Hi-trap Q HP anion exchange column (GE Healthcare Life Sciences, Marlborough, MA, USA). Extracts from the different fractions were then migrated by SDS-PAGE and we identified the fractions containing α-syn after Coomassie staining. The selected fractions were then collected and dialyzed against PBS buffer (GE Healthcare Life Sciences, Marlborough, MA, USA). We then measured the final concentration of purified recombinant α-syn using a NanoDrop 2000 (Thermofisher Scientific, Waltham, MA, USA) and concentrated if needed. Aliquots were stored at −80 °C until use. For fibril assembly, purified recombinant α-syn was thawed and diluted to 5 mg/mL in PBS and under continuous shaking at 1000 rpm at 37 °C in a Thermomixer (Eppendorf, Hamburg, Germany) for 7 days. Fibrils were aliquoted and frozen at −80 °C until use.

Before injection, human α-syn fibrils (PFFs, 5 µg/µL) were thawed at RT and sonicated at RT as previously described in Graham et al., 2018 [10]. Human α-syn monomers (huMonomers) were thawed and we collected the supernatant after ultracentrifugation at 100,000 *g* for 30 min. We injected mice stereotactically with PFFs (*n* = 20) or huMonomers (*n* = 20) (0.8 µL, 5 µg/µL) in the OB (unilateral) of 2 months-old wild type mice as previously described [7,40]. Two mice injected with huMonomers were euthanized after developing severe dermatitis, unrelated to the surgical procedure.

We imaged the fibrils post-sonication by transmission electron microscopy to check the morphology of the fibrils. Human fibrils (after sonication) were diluted to 0.1 µg/µL into sterile PBS and negatively stained with 2% uranyl formate (Electron Microscopy Science, Hatfield, PA, USA, ref #22400). Grids were imaged using a FEI Tecnai G2 Spirit TWIN transmission electron microscope (FEI Company, Hillsboro, OR, USA) at 120 kV (Figure S1).

### 4.3. Serum Collection

Serum samples were acquired as previously described by our group [10]. Three months post-injection, mice were deeply anesthetized with sodium pentobarbital and we collected blood at final bleed by cardiac puncture in BD red top–vacutainer tubes. We kept the tubes at RT for 20–30 min to allow blood clot formation and then centrifuged them at 4500 *g* for 10 min at 15 °C. The serum was collected and transferred to pre-cooled vials, vortexed, aliquoted and frozen on crushed dry ice. Samples were then stored at −80 °C.

### 4.4. Bile Acid Quantification

Bile acids were analyzed using the Biocrates^®^ Bile Acids Kit (Biocrates Life Science AG, Innsbruck, Austria) as described by our group previously [22]. In brief, data were acquired on a Waters TQ-S spectrometer coupled with an Acquity I-Class ultra-pressure liquid chromatography (UPLC) system. All serum specimens were acquired in accordance with the protocol as described in the Bile Acids kit manual. All data analysis was completed using the Biocrates MetIDQ software and TargetLynx (Waters, Milford, MA, USA).

### 4.5. Statistical Analysis

All data were analyzed using MetaboAnalyst (v4.0) [41]. A Wilcoxon–Mann–Whitney U-test was performed on all data acquired to determine whether there were any significantly different metabolites between prodromal PD model mice and age-matched controls injected with HuMonomers (*p* < 0.05; *q*-value < 0.05). Bonferroni-corrected p-values were used to correct for multiple comparisons.

Prior to logistic regression analyses, all data were normalized to the sum and autoscaled. To select the predictor variables used in the logistic regression analyses, Least Absolute Shrinkage and Selection Operator (LASSO) and stepwise variable selection were utilized for optimizing all the model components [42]. A k-fold cross-validation (CV) technique was used to show that the models were not over fit and to assess potential predictive accuracy in an independent sample [43]. Area under the curve (AUC (95% confidence interval)), sensitivity and specificity values were calculated to estimate the performance of the logistic regression and ROC analyses. 

## Figures and Tables

**Table 1:** Results of the univariate analyses for bile acids measured in serum from mice injected with HuMonomers and PFFs. *p*-Values were calculated using the Wilcoxon–Mann–Whitney test. LOD-Limit of detection; LLOQ-Lower limit of quantification. Those bile acids highlighted in bold are considered statistically significantly different (*p* < 0.05; *q* < 0.05).

HMDB#	Name	Mean (SD) of HuMonomer	Mean (SD) of PFF	*p*-Value	*q*-Value (FDR)	Fold Change	LOD	LLOQ
HMDB0000619	Cholic Acid	11.09 (20.89)	10.12 (18.99)	0.24	0.39	1.10	0.004	0.03
HMDB0000518	Chenodeoxycholic acid	0.89 (1.22)	0.77 (1.53)	0.06	0.19	1.15	0.005	0.02
HMDB0000626	Deoxycholic acid	1.63 (2.07)	1.52 (2.61)	0.20	0.39	1.08	0.005	0.02
HMDB0000138	Glycocholic acid	0.07 (0.07)	0.06 (0.06)	0.67	0.85	1.14	0.003	0.03
HMDB0000637	Glycochenodeoxycholic acid	0.06 (0.14)	0.07 (0.14)	0.19	0.39	−1.07	0.01	0.02
HMDB0000631	Glycodeoxycholic acid	0.66 (0.77)	0.35 (0.46)	0.37	0.55	1.90	0.01	0.01
HMDB0000733	Hyodeoxycholic acid	0.65 (0.51)	0.44 (0.52)	0.04	0.16	1.47	0.005	0.02
HMDB0000761	Lithocholic acid	0.10 (0.13)	0.10 (0.15)	0.76	0.85	−1.04	0.002	0.01
HMDB0000506	Alpha-Muricholic acid	0.83 (1.42)	0.65 (1.23)	0.06	0.19	1.28	0.007	0.01
HMDB0000415	Beta-Muricholic acid	7.49 (10.54)	5.72 (8.760)	0.09	0.23	1.31	0.008	0.02
**HMDB0000364**	**Omega-Murichoclic acid**	**4.58 (2.04)**	**2.00 (2.03)**	**<0.0001**	**0.01**	**2.28**	0.007	0.01
HMDB0000036	Taurocholic acid	11.02 (17.81)	9.20 (20.59)	0.93	0.98	1.20	0.008	0.02
HMDB0000951	Taurochenodeoxycholic acid	0.75 (1.22)	0.79 (1.56)	0.99	0.99	−1.05	0.005	0.01
HMDB0000896	Taurodeoxycholic acid	0.29 (0.23)	0.35 (0.42)	0.74	0.85	−1.22	0.001	0.01
HMDB0000722	Taurolithocholic acid	0.01 (0.02)	0.02 (0.03)	0.40	0.55	−1.41	0.001	0.01
HMDB0000932	Tauromuricholic acid (sum of α and β)	1.07 (1.85)	0.42 (0.96)	0.22	0.39	2.52	0.001	0.01
**HMDB0000874**	**Tauroursodeoxycholic acid**	**1.67 (2.71)**	**0.12 (0.12)**	**<0.0001**	**<0.001**	**14.14**	0.001	0.01
**HMDB0000946**	**Ursodeoxycholic acid**	**0.55 (0.58)**	**0.03 (0.05)**	**<0.0001**	**<0.0001**	**17.55**	0.001	0.02

**Table 2:** Logistic Regression Model—Summary of Each Feature.

	Estimate	Std. Error	*z* Value	Pr (>|z|)	Odds
(Intercept)	−0.893	2.857	−0.313	0.755	-
TLCA	11.152	7.264	1.535	0.125	69,675.46
GCDCA	8.917	9.571	0.932	0.352	7455.77
TUDCA	−18.221	7.762	−2.347	0.019	0

**Table 3:** The performance values for the logistic regression model.

	AUC	Sensitivity	Specificity
Training/Discovery	0.992 (0.985~0.998)	0.958 (0.929~0.986)	0.944 (0.907~0.982)
10-fold Cross-Validation	0.906 (0.777~1.000)	0.952 (0.952~1.000)	0.938 (0.819~1.000)

## Supplementary Materials

The following are available online at http://www.mdpi.com/2218-1989/8/4/71/s1, Figure S1: Sonicated PFFs stained by uranyl formate, imaged by transmission electron microscopy to confirm their fibrillary nature.
