# Supplementary material for: Metabolomic Profiling of Bile Acids in an Experimental Model of Prodromal Parkinson’s Disease
Source: Metabolites. 2018 Oct 31;8(4):71. doi: 10.3390/metabo8040071 (PMC6316593; doi:10.3390/metabo8040071)
Supplement: Supplementary file 1 [file metabolites-08-00071-s001.pdf]

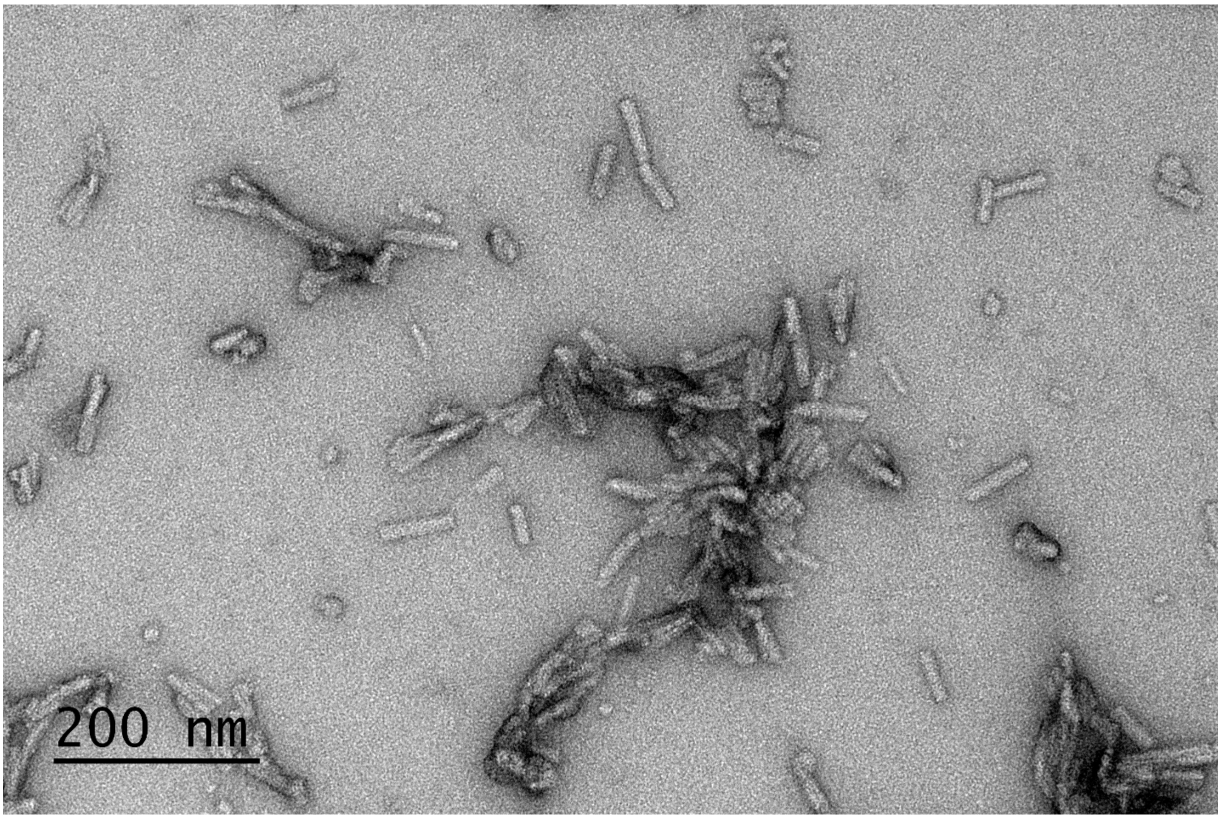

Figure S1. Sonicated PFFs stained by uranyl formate, imaged by transmission electron microscopy to confirm their fibrillary nature.
